# Non-canonical NFκB activation promotes chemokine expression in podocytes

**DOI:** 10.1038/srep28857

**Published:** 2016-06-29

**Authors:** Lara Valiño-Rivas, Laura Gonzalez-Lafuente, Ana B. Sanz, Marta Ruiz-Ortega, Alberto Ortiz, Maria D. Sanchez-Niño

**Affiliations:** 1 IIS-Fundación Jiménez Díaz-Universidad Autónoma de Madrid and Fundación Renal Iñigo Alvarez de Toledo-IRSIN, Madrid, Spain; 2 REDINREN, Madrid, Spain

## Abstract

TNF-like weak inducer of apoptosis (TWEAK) receptor Fn14 is expressed by podocytes and Fn14 deficiency protects from experimental proteinuric kidney disease. However, the downstream effectors of TWEAK/Fn14 in podocytes are poorly characterized. We have explored TWEAK activation of non-canonical NFκB signaling in cultured podocytes. In cultured podocytes, TWEAK increased the expression of the chemokines CCL21, CCL19 and RANTES in a time-dependent manner. The inhibitor of canonical NFκB activation parthenolide inhibited the CCL19 and the early RANTES responses, but not the CCL21 or late RANTES responses. In this regard, TWEAK induced non-canonical NFκB activation in podocytes, characterized by NFκB2/p100 processing to NFκB2/p52 and nuclear migration of RelB/p52. Silencing by a specific siRNA of NIK, the upstream kinase of the non-canonical NFκB pathway, prevented CCL21 upregulation but did not modulate CCL19 or RANTES expression in response to TWEAK, thus establishing CCL21 as a non-canonical NFκB target in podocytes. Increased kidney Fn14 and CCL21 expression was also observed in rat proteinuric kidney disease induced by puromycin, and was localized to podocytes. In conclusion, TWEAK activates the non-canonical NFκB pathway in podocytes, leading to upregulation of CCL21 expression. The non-canonical NFκB pathway should be explored as a potential therapeutic target in proteinuric kidney disease.

Chronic kidney disease (CKD) is one the three causes of death that most increased worldwide from 1990 to 2013^1^,^2^. CKD is now categorized based on glomerular filtration rate and degree of albuminuria, since albuminuria, a marker of podocyte injury, is a key risk factor for death and for progression of CKD. Proteinuric kidney diseases, such as diabetic kidney disease and chronic glomerulopathies, remain the most frequent causes of CKD, despite the availability of antiproteinuric agents such as renin-angiotensin system blockers^3^. Thus, there is an increasing interest in understanding the molecular mechanisms of podocyte injury or activation to express inflammatory mediators that may contribute to proteinuric kidney disease. Recently, functional *in vivo* studies identified the TNF superfamily cytokine Tumor necrosis factor-like weak inducer of apoptosis (TWEAK, Apo3L or TNFSF12) as a key contributor to proteinuric kidney disease in the context of immune-complex deposition (lupus nephritis, anti-glomerular basement membrane disease) and in the absence of immune-mediated injury (protein overload)^4^,^5^,^6^,^7^,^8^. Fn14-knockout MRL-lpr/lpr mice were protected from glomerular injury and glomerular podocytes were preserved^5^. Based on this preclinical data, clinical trials are testing the hypothesis that anti-TWEAK neutralizing antibodies are nephroprotective in human lupus nephritis^4^. However, TWEAK actions on podocytes have been poorly characterized. TWEAK signaling directly damaged barrier function and increased filtration through podocyte monolayers^5^, induced cell motility^9^ and promoted inflammatory responses in podocytes^8^. In this regard, the podocyte contribution to glomerular inflammation has recently been emphasized^10^,^11^,^12^. Thus, we have now explored further actions of TWEAK in podocytes. Specifically, we have focused on the regulation of the activation of the pro-inflammatory transcription factor nuclear factor-kappa B (NFκB) in podocytes^13^,^14^, since unravelling this pathway may provide new possibilities for development of therapeutic approaches for proteinuric kidney disease. Like TNF, TWEAK activates canonical NFκB signaling in diverse cell types, including podocytes^8^,^15^,^16^. Unlike TNF, TWEAK promotes the non-canonical activation of NFκB in splenocytes and tubular cells^14^,^17^. In tubular cells, TWEAK-induced non-canonical NFκB activation led to expression of CCL21, a chemokine previously described as a transcriptional target of non-canonical NFκB activation in splenocytes^18^. However, little is known of non-canonical NFκB activation in podocytes (reviewed in ref. 14). Interestingly, podocyte CCL21 was reported to contribute to the podocyte-mesangial cell crosstalk by activating CCR7 receptors in mesangial cells and promoting mesangial cell survival, proliferation, migration and matrix adhesion through activation of glycogen synthase kinase-3 (GSK-3), protein kinase B (PKB/Akt) and integrin-linked kinase^19^,^20^,^21^. While CCL21-induced mesangial cell proliferation was initially hypothesized to contribute to glomerular homeostasis, the hypothesis was not tested and several glomerulonephritis are characterized by mesangial proliferation. Furthermore, kidney CCL21 expression was associated to recurrence of original nephropathy among renal transplant patients^22^ and to acute rejection in zero-hour biopsies from deceased donor kidneys^23^, and was hypothesized to facilitate alloreactive immune responses in renal transplant recipients^24^. Moreover, anti-CCL21 antibodies reduced kidney fibrosis and inflammation through inhibition of fibrocyte recruitment^25^. Thus, functional *in vivo* preclinical studies are consistent with a detrimental role of CCL21 in kidney fibrosis and inflammation. CCL21 expression by podocytes could thus be chemotactic or elicit detrimental biological responses in mesangial or tubular cells. However, the factors regulating CCL21 expression in podocytes have not been characterized.

We have now explored whether TWEAK modulates CCL21 expression in podocytes and the role of non-canonical NFκB activation in the process. Furthermore, we have explored the expression of the TWEAK receptor and CCL21 in experimental proteinuric kidney disease.

## Results

### TWEAK increases CCL21 expression in cultured podocytes and this response is independent from canonical NFκB activation

**Figure 1:**
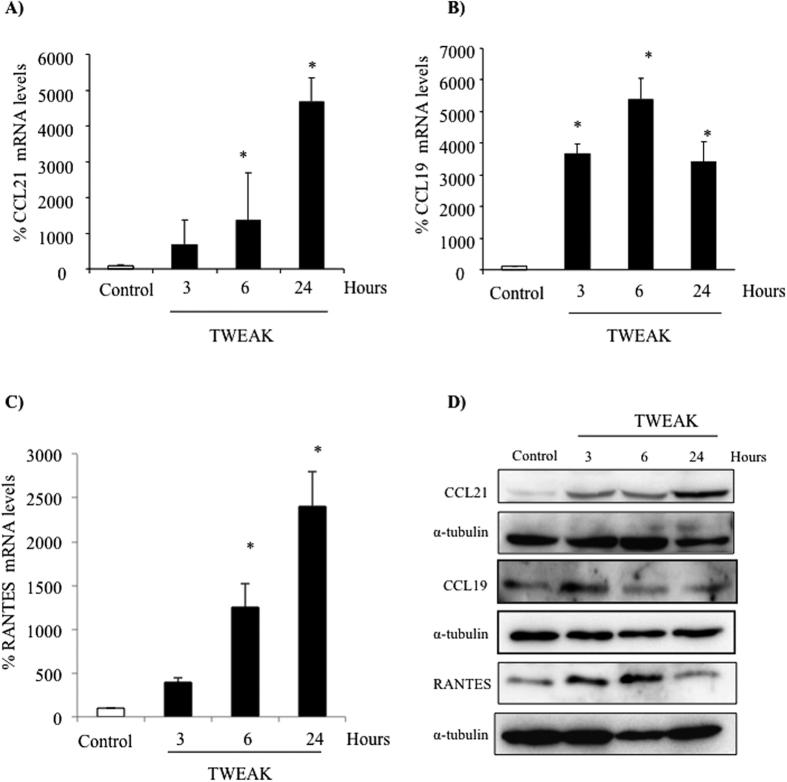
TWEAK upregulates chemokine mRNA and protein expression in podocytes: differences in time-course. Podocytes were treated with 100 ng/mL TWEAK. (**A**) Time-course of CCL21 mRNA induction. *p < 0.004 vs control. (**B**) Time-course of CCL19 mRNA induction. *p < 0.002 vs control. (**C**) Time-course of RANTES mRNA induction. *p < 0.003 vs control. Expression of mRNA was assessed by real time RT-PCR. Mean ± SEM of three independent experiments. (**D**) Representative Western blot of CCL21, CCL19 and RANTES protein in cell lysates.

The time-course of TWEAK-induced chemokine expression differed between cultured podocytes and tubular cells^15^,^17^. This may imply differential involvement of different NFκB activation pathways. TWEAK-induced mRNA expression of the non-canonical NFκB target CCL21 progressively increased over 24 h in podocytes (Fig. 1A) as was observed for tubular cells^17^. TWEAK also increased the expression of CCL19 mRNA, which is also a target of non-canonical NFκB activation in splenocytes^26^,^27^ (Fig. 1B). However, unlike the similar time-course observed for TWEAK-induced CCL21 and CCL19 expression in tubular cells^17^, the time-course of CCL19 and CCL21 expression differed in podocytes. Thus, CCL19 expression peaked at 6 h and was already decreasing at 24 h (Fig. 1B). TWEAK also increased RANTES mRNA expression progressively for up to 24 h (Fig. 1C). This is in contrast to tubular cells, in which TWEAK-induced RANTES expression peaks at 6 h, as expected for a canonical NFκB target, and then decreases^15^. Increased protein levels in cell lysates was also noted (Fig. 1D).

**Figure 2:**
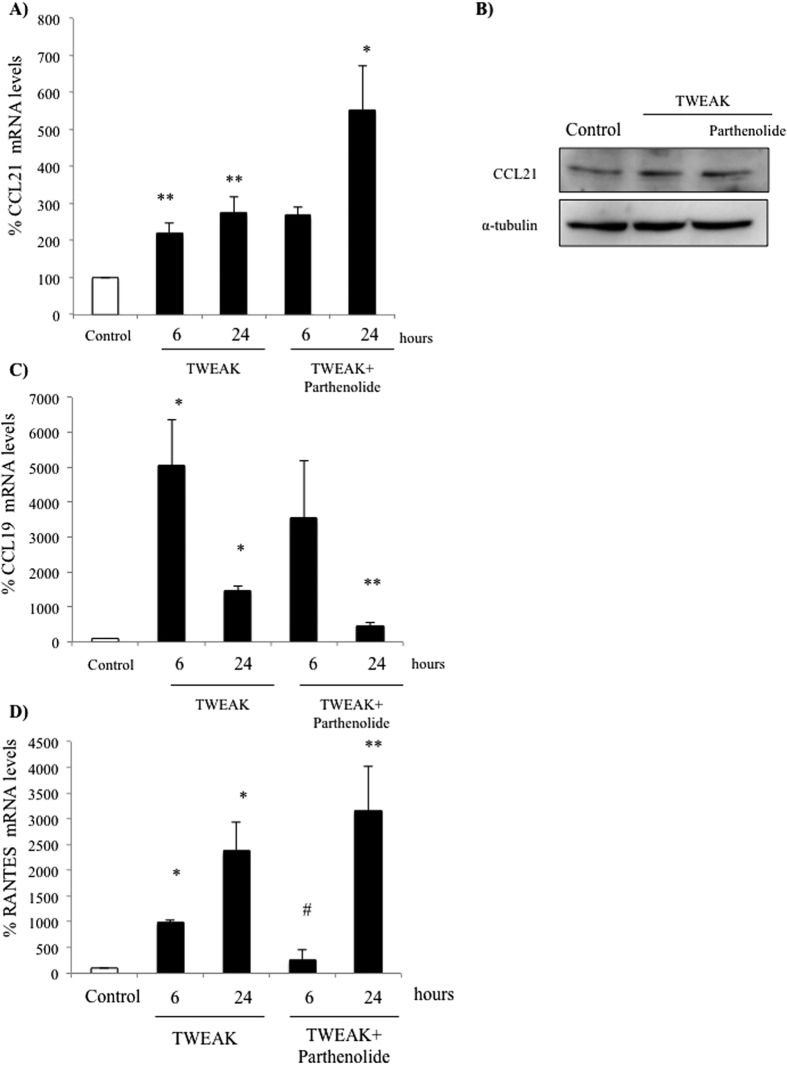
Targeting canonical NFκB activation modulates RANTES and CCL19 but not CCL21 expression in podocytes. Podocytes were treated with 100 ng/mL TWEAK. (**A**) CCL21 mRNA. **p < 0.001 vs control. *p < 0.01 vs TWEAK 24 h. (**B**) CCL21 protein at 24 h (Western blot). (**C**) CCL19 mRNA.*p < 0.001 vs control. **p < 0.01 vs TWEAK 24 h. (**D**) RANTES mRNA. *p < 0.01vs Control, **p < 0.009 vs TWEAK 24 h. Expression of mRNA was assessed by real time RT-PCR and protein by Western blot. Mean ± SEM of three independent experiments.

Parthenolide inhibits IκBα degradation and RelA nuclear translocation and thus, canonical NFκB activation^15^,^28^. In previous studies, we observed that parthenolide inhibited RelA nuclear translocation induced by TWEAK in podocytes as well as the expression of the canonical RelA target gene MCP-1^8^. However, parthenolide did not prevent CCL21 mRNA or protein up-regulation induced by TWEAK (Fig. 2A,B) suggesting that RelA does not mediate CCL21 transcription. By contrast, parthenolide did prevent the upregulation of CCL19 mRNA in podocytes (Fig. 2C), suggesting the involvement of different pathways for NFκB activation in the regulation of both chemokines in podocytes, in contrast to prior reports in splenocytes^18^,^26^. Parthenolide prevented the early, but not the late increase in RANTES mRNA expression (Fig. 2D), suggesting the contribution of canonical but also of parthenolide-resistant pathways to TWEAK-induced RANTES upregulation in podocytes. Similar results were observed using the NF-κB inhibitor BAY 11-7085 (Suppl. Fig. 1).

### TWEAK induces non-canonical NFκB activation in cultured podocytes

**Figure 3:**
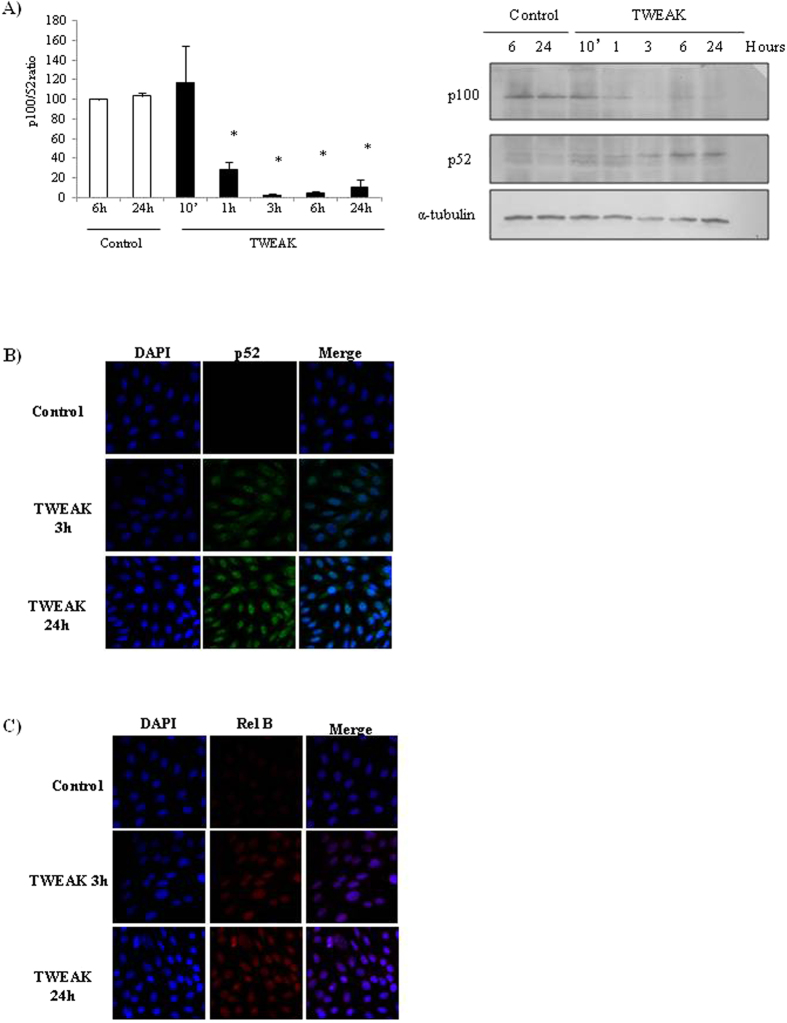
TWEAK induces NFκB2/p100 processing to NFκB2/p52 and nuclear translocation of NFκB2/p52 and RelB in cultured podocytes. (**A**) Western blot and quantification. Note disappearance of p100 from 1 hour onwards and concomitant appearance of the p52 band. Mean ± SEM of three independent experiments. *p < 0.05 vs control. (**B**) Confocal microscopy of NFκB2 (green), nuclei counterstained with DAPI (blue); and (**C**) Confocal microscopy of RelB (red), nuclei counterstained with DAPI (blue). NFκB2 and RelB progressively increased in podocyte nuclei from 3 to 24 hours of TWEAK stimulation.

**Figure 4:**
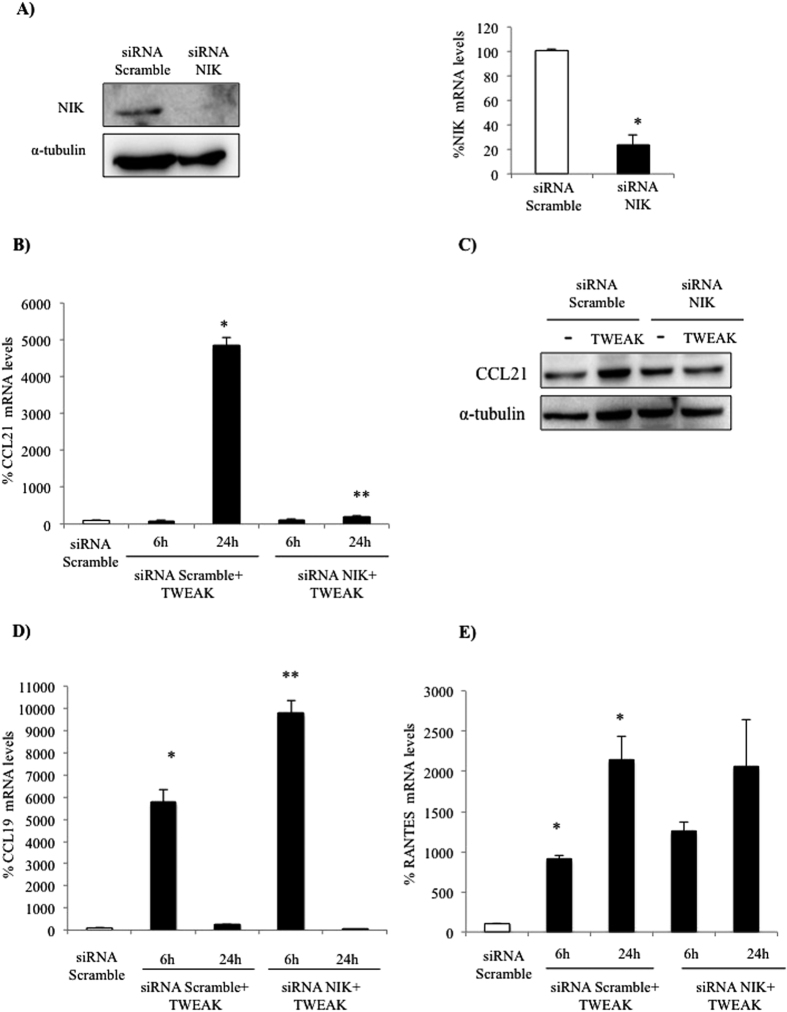
NIK targeting prevents TWEAK-induced CCL21 but not CCL19 expression in cultured podocytes. (**A**) NIK siRNA downregulates NIK protein and mRNA expression. *p < 0.005 vs Scramble siRNA. (**B**) NIK siRNA prevents the upregulation of CCL21 mRNA in response to TWEAK. (**C**) NIK siRNA prevents the upregulation of CCL21 protein in response to TWEAK at 24 h. (**D**) NIK siRNA does not prevent the upregulation of CCL19 mRNA in response to TWEAK. (**E**) NIK siRNA does not prevent the upregulation of RANTES mRNA in response to TWEAK. Expression of mRNA was assessed by real time RT-PCR and protein by Western blot. Mean ± SEM of three independent experiments, *p < 0.005 vs Scramble siRNA, **p < 0.005 vs Scramble siRNA+TWEAK.

In previous studies we observed that TWEAK induced a sustained increase in NFκB DNA-binding activity in podocytes that peaked at 24 h as assessed by electrophoretic mobility shift assay (EMSA)^8^. This sustained increase is consistent with non-canonical NFκB activation, since canonical NFκB is a transient phenomenon, but non-canonical NFκB activation in response to TWEAK in other epithelial cells, such as tubular cells, increases progressively up to 24 h^13^. The non-canonical NFκB pathway requires activation of NFκB-inducing kinase (NIK), that phosphorylates IKKα and serves as a docking molecule that recruits IKKα to NFκB2 p100, facilitating NFκB2 p100 ubiquitination and subsequent proteasomal processing into the mature NFκB2 p52 subunit, allowing RelB/p52 complexes to enter the nucleus^14^. TWEAK induces NFκB2 p100 processing to NFκB2 p52 (Fig. 3A) as well as p52/RelB nuclear translocation in cultured podocytes in a time-dependent manner (Fig. 3B,C). These results indicate that TWEAK induces non-canonical, sustained NFκB activation in podocytes. To explore the role of non-canonical NFκB activation in the regulation of TWEAK-induced chemokine expression, we silenced NIK using a specific siRNA (Fig. 4A). NIK silencing prevented CCL21 upregulation in cultured podocytes (Fig. 4B,C) but did not modulate CCL19 or RANTES expression (Fig. 4D,E). This suggests that CCL21 is a non-canonical NFκB pathway target in podocytes, but other chemokines are not.

### Increased podocyte CCL21 in experimental proteinuric kidney disease

**Figure 5:**
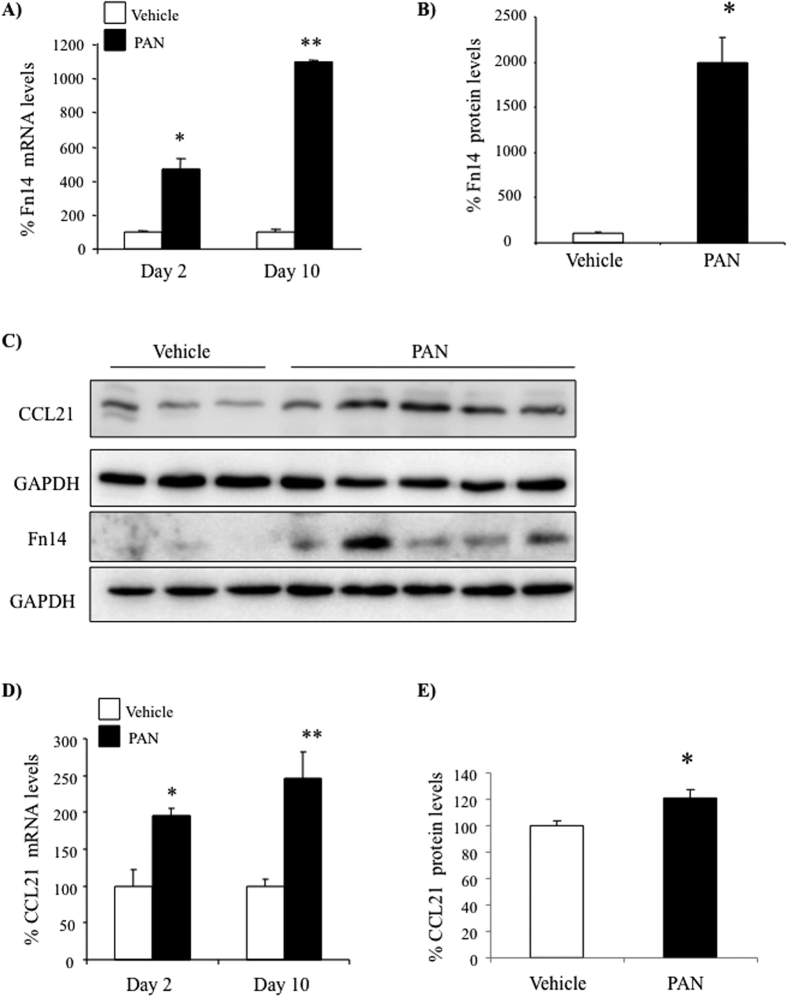
Fn14 and CCL21 mRNA and protein upregulation in PAN nephrosis. (**A**) Whole kidney Fn14 mRNA expression. qRT-PCR. (**B**) Whole kidney Fn14 protein expression. Quantification of Western blot. (**C**) Whole kidney Fn14 and CCL21. Western blot. (**D**) Whole kidney CCL21mRNA expression. qRT-PCR. (**E**)Whole kidney CCL21protein expression. Quantification of Western blot. Mean ± SEM of 5 rats, *p < 0.05 vs vehicle, **p < 0.001 vs vehicle.

**Figure 6:**
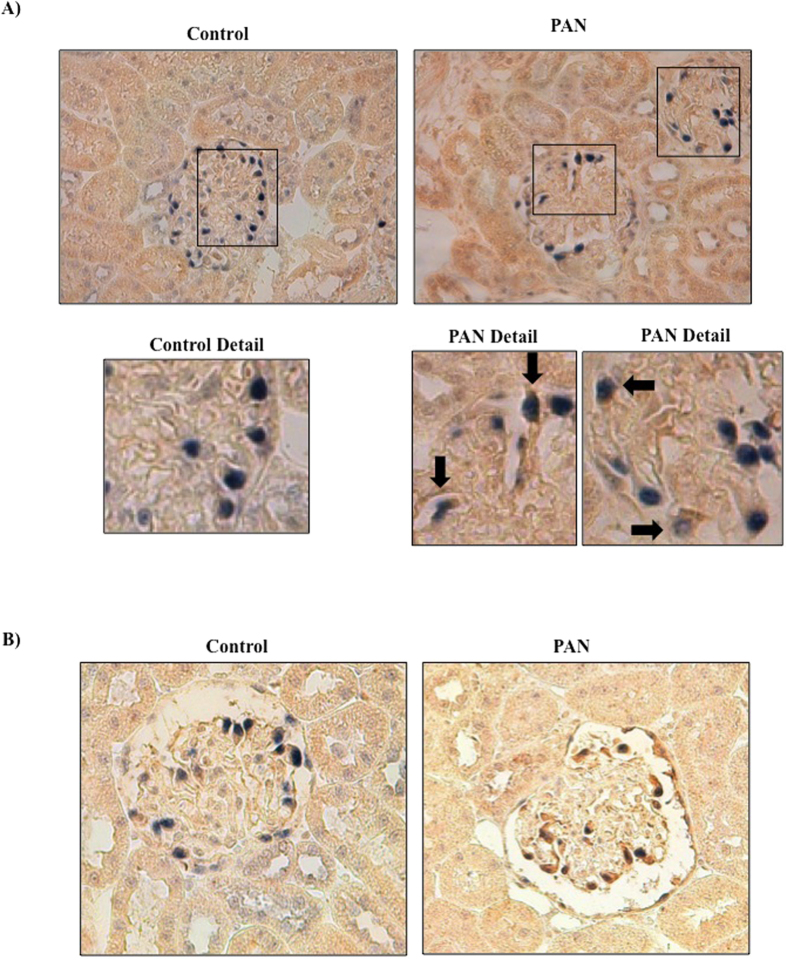
Fn14 and CCL21 upregulation in PAN nephrosis. Immunohistochemistry. (**A**) Fn14 and (**B**) CCL21 immunohistochemistry 10 days following PAN or vehicle injection. Note expression of Fn14 and CCL21 by WT1 positive (blue) podocytes (arrow) in PAN-treated rats (arrows). Original magnification x400. Detail x1000. Images representative of 6/7 animals per group.

We next explored the expression of the chemokine identified as a non-canonical NFκB target, CCL21, during podocyte injury and by podocytes *in vivo*. For assessment of CCL21 expression in proteinuric kidney disease we chose a non-immunological model of podocyte injury, rat PAN nephrosis, since it is directly induced by a podocyte toxin, does not require activation of the immune system and is representative of human focal segmental glomerulosclerosis, a common human proteinuric nephropathy^29^,^30^. Systemic PAN administration causes podocyte injury in rats, leading to increased urinary protein excretion by day 2 and full-blown nephrotic syndrome at day 10. Increased whole kidney TWEAK receptor (Fn14) mRNA (Fig. 5A) and protein (Fig. 5B,C) expression was noted in PAN-injected rats 2 and 10 days post-injection. Moreover, CCL21 mRNA (Fig. 5D) and protein (Fig. 5C,E) was also increased, following a similar time-course. Immunohistochemistry confirmed Fn14 and CCL21 protein expressing cells co-localized with the podocyte-specific maker WT-1 in glomeruli of PAN-injected rats (Fig. 6 and Suppl. Fig. 2).

To better understand the relationship between podocyte injury and expression of inflammatory molecules, we tested the regulation of Fn14 and CCL21 expression by PAN or by high glucose in cultured podocytes. Either a cytotoxic concentration of PAN or high glucose levels increased Fn14 and CCL21 mRNA (Suppl. Fig. 3) expression in cultured podocytes.

## Discussion

TWEAK had previously been shown to have a proinflammatory effect dependent on canonical NFκB activation in podocytes, leading to the synthesis of the MCP-1 chemokine^8^,^31^. We now show that TWEAK also activates the non-canonical NFκB pathway in podocytes, promoting the expression of the chemokine CCL21 that, as Fn14, is upregulated *in vivo* in the course of nephrotoxic podocyte injury.

NFκB activates the transcription of different genes with specificity and kinetics that vary in a gene-, stimulus- and cell-specific manner. Delayed kinetics of gene transcription may be due to involvement of the non-canonical NFκB pathway or to decreased DNA accessibility to the canonical NFκB pathway. The later has been described for RANTES in tubular cells and other cell types^13^,^15^. We have now focused on understanding the regulators and targets of non-canonical NFκB activation in podocytes in response to TWEAK, a cytokine that promotes glomerular injury in experimental animals and is currently the target of clinical trials in human glomerular disease.

Some actions of TWEAK in podocytes had been previously characterized, including damage to the barrier function^5^, motility^9^ and inflammatory responses as a consequence of canonical NFκB activation leading to MCP-1 synthesis^8^. These observations may underlie the causative role of TWEAK in glomerular injury^4^,^32^. Interestingly, in podocytes TWEAK induced sustained NFκB activation as assessed by EMSA^8^, although the molecular basis for the sustained NFκB activation was not explored. We now provide evidence that in podocytes TWEAK recruits sequentially the canonical and non-canonical pathways of NFκB activation, resulting in the transcription of different sets of chemokines. Initially canonical activation of RelA promotes the release of MCP-1^8^, and as identified here, the gene expression of RANTES and CCL19, followed by activation of the non-canonical NFκB pathway requiring NIK, NFκB2 p100 processing and migration of NFκB2 p52/RelB to nuclei that promotes synthesis of CCL21. The transient nature of chemokine protein expression in cell lysates (3 h for CCL19, 3–6 h for RANTES) may be due to either posttranscriptional regulatory mechanisms or secretion of the chemokine into the cell culture medium. Thus, CCL21 is identified as a TWEAK- and non-canonical NFκB pathway-regulated gene in podocytes. In this regard, podocytes differ from splenocytes, which were reported to require non-canonical NFκB activation by LTβR ligation to upregulate both CCL21 and CCL19^33^.

TWEAK induced non-canonical NFκB activation in fibroblasts^34^ and in kidney tubular cells^17^, but it was unknown whether the pathway is active in podocytes. Information on non-canonical NFκB targets has been detailed for lymphoid cells. Thus, LTβR activation of the non-canonical NFκB pathway induces CCL21, CCL19, CXCL12, CXCL13, and TNFSF13b (BAFF/BLYSS) expression in the spleen^33^. Of these potential NFκB2 targets, we chose CCL19 and CCL21 for further study in podocytes, together with RANTES, a representative of genes with a delayed response to canonical NFκB activation in other cell types. Only CCL21 was regulated by the non-canonical NFκB target in podocytes. TWEAK-induced increased CCL21 mRNA was delayed with respect to other chemokines and persistent. This contrasts to the earlier peaks of MCP-1^8^, RANTES and CCL19 which were abolished by RelA inhibitors. The delayed induction of CCL21 is dependent on the delayed activation of the non-canonical NFκB pathway that requires NIK. To the best of our knowledge CCL21 is the first non-canonical NFκB2 target identified in podocytes. The *in vivo* observation of increased CCL21 and Fn14 expression in experimental proteinuric kidney disease suggests that this might be a clinically relevant observation.

**Figure 7:**
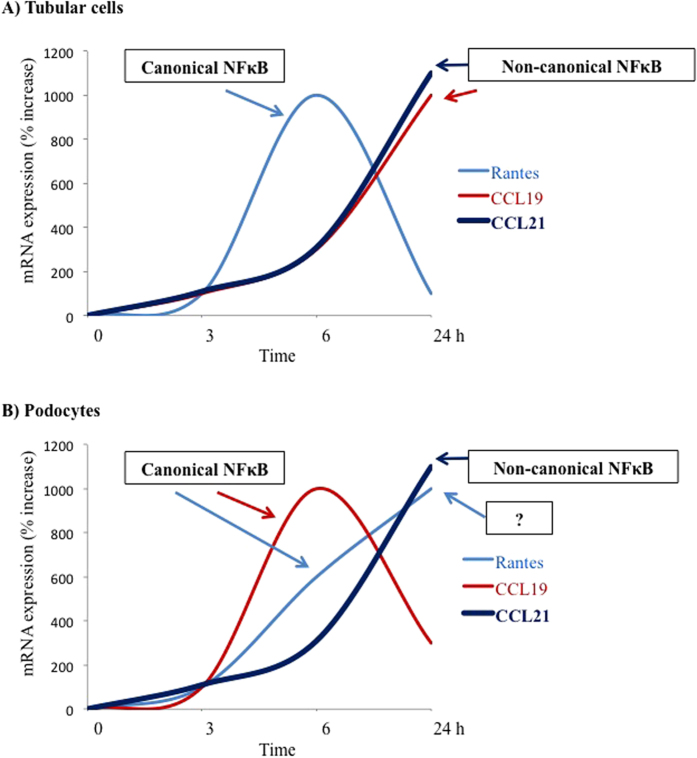
Differential involvement of NFκB activation pathways in the regulation of chemokine expression in renal tubular cells and podocytes. In splenocytes, both CCL19 and CCL21 are transcriptional targets of the non-canonical pathway for NFκB activation involving NFκB2/RelB heterodimers^18^. (**A**) Murine renal tubular cells^15^,^17^. TWEAK-induced RANTES expression is inhibited by parthenolide, suggesting canonical NFκB activation. By contrast, CCL21 expression is prevented by NIK siRNA, but not by parthenolide. While not specifically studied by functional inhibitors, the time-course of CCL19 expression and the fact that CCL19 was upregulated by TWEAK but not by TNF suggests that CCL19 is a transcriptional target for non-canonical NFκB activation in tubular cells. (**B**) Podocytes. Note the different time-course of TWEAK-induced RANTES and CCL19 mRNA expression between tubular cells and podocytes. The increased CCL19 mRNA and the early increase in RANTES mRNA is inhibited by parthenolide, suggesting dependence on canonical NFκB activation. CCL21 is the only chemokine that depends on non-canonical NFκB activation in splenocytes, tubular cells and podocytes. The late increase of RANTES mRNA expression in podocytes could not be inhibited by targeting the canonical or non-canonical pathways suggesting NFκB-independence or activation of alternative NFκB pathways^13^,^15^.

Interestingly, differences were apparent between the pathways for NFκB activation and the time-course of chemokine expression between podocytes and tubular cells and splenocytes^15^,^17^,^26^,^27^ (Fig. 7). This argues for podocyte-specific mechanism regulating chemokine expression and for the need to understand the precise molecular mechanisms involved in the regulation of podocyte inflammatory responses, since not all observations in other renal cell types apply to podocytes. This offers the opportunity for differential manipulation of inflammatory responses in a cell type-specific fashion in the kidneys.

CCL21 is a high affinity ligand for chemokine receptor 7 (CCR7). Activation of CCR7 is chemotactic for thymocytes, T cells, mature dendritic cells, and, to a lesser extent, B cells^35^. CCL21 mediates homing of lymphocytes to secondary lymphoid organs, attracts CCR7-positive fibrocytes to injured kidneys and induces chemotaxis and proliferation of mesangial cells^19^,^25^. In this regard, podocytes expression of CCL21 activated CCR7 receptors in mesangial cells^19^,^20^,^21^. According to the Nephromine (http://www.nephromine.org/) database that integrates transcriptomics data from several published sources, glomerular CCL21 mRNA was increased in human collapsing FSGS (fold change 1.591, p = 0.045), diabetic nephropathy (1.477, p = 0.023), lupus nephritis (1.316, p = 0.034) and IgA nephropathy (1.137, p = 0.040) and a trend was observed in FSGS (1.386, p = NS) when compared to normal glomeruli (http://www.nephromine.org/; accessed September 1, 2015)^36^,^37^,^38^,^39^. This suggests that experimental findings have implications for human proteinuric kidney disease. However, the factors that regulate podocyte CCL21 expression had not been identified.

In conclusion, TWEAK is an activator of the non-canonical NFκB pathway in podocytes, and the chemokine CCL21 is activated through this pathway while CCL19 or RANTES are not. To our knowledge this is the first time that stimuli modulating non-canonical NFκB activation and gene targets have been described in podocytes. Activation of the non-canonical NFκB pathway may contribute to the deleterious effect of TWEAK in proteinuric kidney disease, as Fn14 knockout mice were protected from proteinuric kidney injury of immune and non-immune origin^5^,^8^ and should be explored as a therapeutic target itself.

## Material and Methods

### Cells and reagents

Conditionally immortalized mouse podocytes were a kind gift by Peter Mundel and were cultured as described^40^. Podocytes were propagated on type I collagen (Biochrom, Berlin, Germany) at 33 °C in the presence of 10 U/mL mouse recombinant interferon (IFN-γ) (Immugenex, Los Angeles, CA) (permissive conditions) to enhance expression of a thermosensitive T antigen. Once cells had reached 70 to 80% confluence, differentiation and a quiescent phenotype was induced by culture under “non-permissive conditions” at 37 °C without IFN-γ for >12 days, resulting in disappearance of T antigen. Culture medium was RPMI 1640 (GIBCO, Grand Island, NY) with 10% heat-inactivated fetal bovine serum (FBS), 2 mM glutamine, 100 U/mL penicillin and 100 μg/mL streptomycin. For experiments cells were cultured in serum-free media 24 hours previous to stimulation and throughout the experiment.

Recombinant human soluble TWEAK (Alexis, Läufelfingen, Switzerland) was used at 100 ng/ml based on prior TWEAK dose-response studies in renal cells^8^. The NFκB inhibitors parthenolide (Sigma, St. Louis, MO) and Bay 11-7085 (R&D Systems, Minneapolis, MN) were used at 10 μM based on previous dose-responses studies in cultured podocytes^8^. Puromycin aminonucleoside (PAN) (Sigma, St. Louis, MO) was used at a concentration of 100 μg/mL. In preliminary experiments, this concentration was found to be cytotoxic to podocytes in culture. For high glucose experiments, glucose was added in the media to reach a final concentration of 700 mg/dl versus control media with 200 mg/dl glucose^41^.

### Western blot

Western blots were performed as described previously^41^. Primary antibodies were rabbit polyclonal anti-Fn14 antibody (1:1000, Cell Signaling, Hertfordshire, UK), rabbit polyclonal anti-p100/52 (1:500, Cell Signaling), rabbit polyclonal anti-CCL21 (1:500, Santa Cruz), goat polyclonal anti-CCL19 (1:500, R&D Systems), RANTES (1:500, Acris) and anti-NIK (1:500, Santa Cruz). Since chemokines were assessed in cell lysates, part of the protein would have been expected to be secreted, especially at later time points. Blots were then probed with anti-α-tubulin (1:2000, Sigma) and levels of expression were corrected for minor differences in loading.

### Quantitative reverse transcription-polymerase chain reaction

One μg RNA isolated by Trizol (Invitrogen UK) was reverse transcribed with High Capacity cDNA Archive Kit and real-time PCR was performed on a ABI Prism 7500 PCR system (Applied Biosystems, Foster City, CA) using the DeltaDelta Ct method. Expression levels are given as ratios to GAPDH. Pre-developed primer and probe assays were from Applied^41^.

### siRNA transfection

Cells were seeded at a 3 × 10^5^ in 6-wells plates and transfected with 25 nM NIK siRNA (Santa Cruz), Opti-MEM I Reduced Serum Medium and Lipofectamine 2000 (Invitrogen)^42^. After 18 h, transfected cells were washed and cultured for 24 hours in complete medium, stimulated with 100 ng/mL TWEAK for 24 h and harvested for analysis. This time point was selected from a time-course of decreasing NIK protein expression in response to siRNA. A negative control scrambled siRNA provided by the manufacturer did not reduce NIK protein.

### Immunohistochemistry

Immunohistochemistry was carried out as previously described on paraffin-embedded 5 μm tissue sections^43^. Primary antibodies were rabbit polyclonal anti-CCL21 (1:75), rabbit polyclonal anti-Fn14 (1:50, Santa Cruz) and anti-WT1 (Dako, Denmark). Negative controls included incubation with a non-specific immunoglobulin of the same isotype as the primary antibody. Sections were counterstained with Carazzi’s hematoxylin.

### Confocal microscopy

Cells plated onto Labtek™ slides were fixed in 4% paraformaldehyde and permeabilized in 0.2% Triton X-100 in PBS for 10 min each. After washing in PBS, cells were incubated overnight at 4 °C with rabbit polyclonal anti-p52 or anti-RelB antibody (1:100), followed by incubation with anti-rabbit Alexa Fluor 488 or 633 (1:300, Invitrogen). After washing, cells were mounted in 70% glycerol in PBS, and analyzed with a DM-IRB confocal microscope (Leica DM, Bannockburn, IL)^31^. Nuclei cells were stained with DAPI (Vector Laboratories, Inc., Burlingame, CA) to observe the typical morphological changes.

### Animal model

Two groups of 10-week-old Wistar Kyoto rats (Criffa, Barcelona, Spain) were studied (n = 9/group). Nephrosis was induced by a single i.v. injection of 150 mg/Kg puromycin aminonucleoside (PAN, Sigma)^29^,^30^. Control rats received saline vehicle. Rats were sacrificed at days 2 and 10 after injection. To determine urinary protein level, rats were placed into metabolic cages 0, 2, and 10 days after PAN injection. Twenty-four hour urine samples were collected in brown bottles and total urinary protein was measured. Proteinuria was 4.8 ± 0.9 mg/d in control rats, 8.0 ± 2.0 mg/d in PAN-injected rats at day 2 and 203.7 ± 44.4 mg/d in PAN-injected rats at day 10; and serum creatinine 0.40 ± 0.01 mg/dl in control rats, 0.47 ± 0.03 mg/dl in PAN-injected rats at day 2 and 0.61 ± 0.05 mg/dl in PAN injected rats at day 10.

Kidneys were perfused *in situ* with cold saline before removal. One kidney from each rat was fixed in buffered formalin, embedded in paraffin and used for immunohistochemistry. The other kidney was snap-frozen in liquid nitrogen for RNA and protein studies. Before sacrifice, animals were placed in metabolic cages for 24 h urine collection and albuminuria determination by ELISA, using rat albumin as a standard (Celltrend, Luckenwalde, Germany). All experimental procedures were approved by the Animal Care and Use Committee of the Institution IIS-Fundacion Jimenez Diaz, according to the guidelines for ethical care of the European Community.

### Statistics

Statistical analysis was performed using SPSS 11.0 statistical software. Results are expressed as mean ± SEM. Significance at the p < 0.05 level was assessed by Student´s t test for two groups of data and ANOVA for three of more groups.

## Additional Information

**How to cite this article**: Valiño-Rivas, L. *et al.* Non-canonical NFκB activation promotes chemokine expression in podocytes. *Sci. Rep.* **6**, 28857; doi: 10.1038/srep28857 (2016).

## Supplementary Material

Supplementary Information
