# Supplementary material for: Non-canonical NFκB activation promotes chemokine expression in podocytes
Source: Sci Rep. 2016 Jun 29;6:28857. doi: 10.1038/srep28857 (PMC4926283; doi:10.1038/srep28857)
Supplement: Supplementary Information [file srep28857-s1.pdf]

Non-canonical NFκB activation promotes chemokine expression in podocytes

Lara Valiño-Rivas [1,2], Laura Gonzalez-Lafuente [1,2], Ana B Sanz [1,2], Marta Ruiz-Ortega [1,2], Alberto Ortiz [1,2*], Maria D Sanchez-Niño [1,2*]

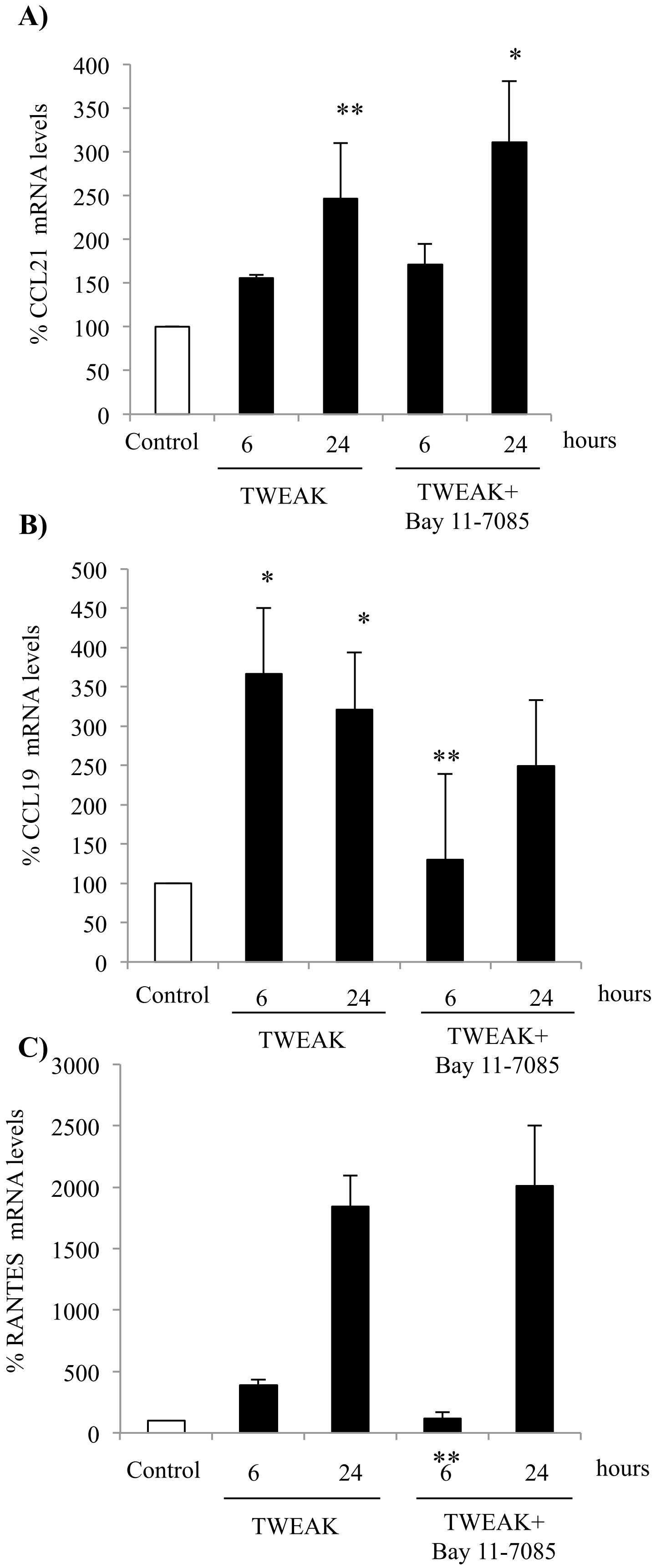

Supplementary figure 1. Targeting canonical NFκB activation modulates RANTES and CCL19 but not CCL21 expression in podocytes. Podocytes were pre-treated with 10 μM Bay 11-7085 and 1 hour later with 100 ng/mL TWEAK. **A)** CCL21 mRNA. **p<0.001 vs control. **B)** CCL19 mRNA.*p<0.001 vs control. **p<0.05 vs TWEAK 6h. C) RANTES mRNA. *p<0.01vs Control, **p<0.009 vs TWEAK 6h. Expression of mRNA was assessed by real time RT-PCR. Mean ± SEM of three independent experiments.

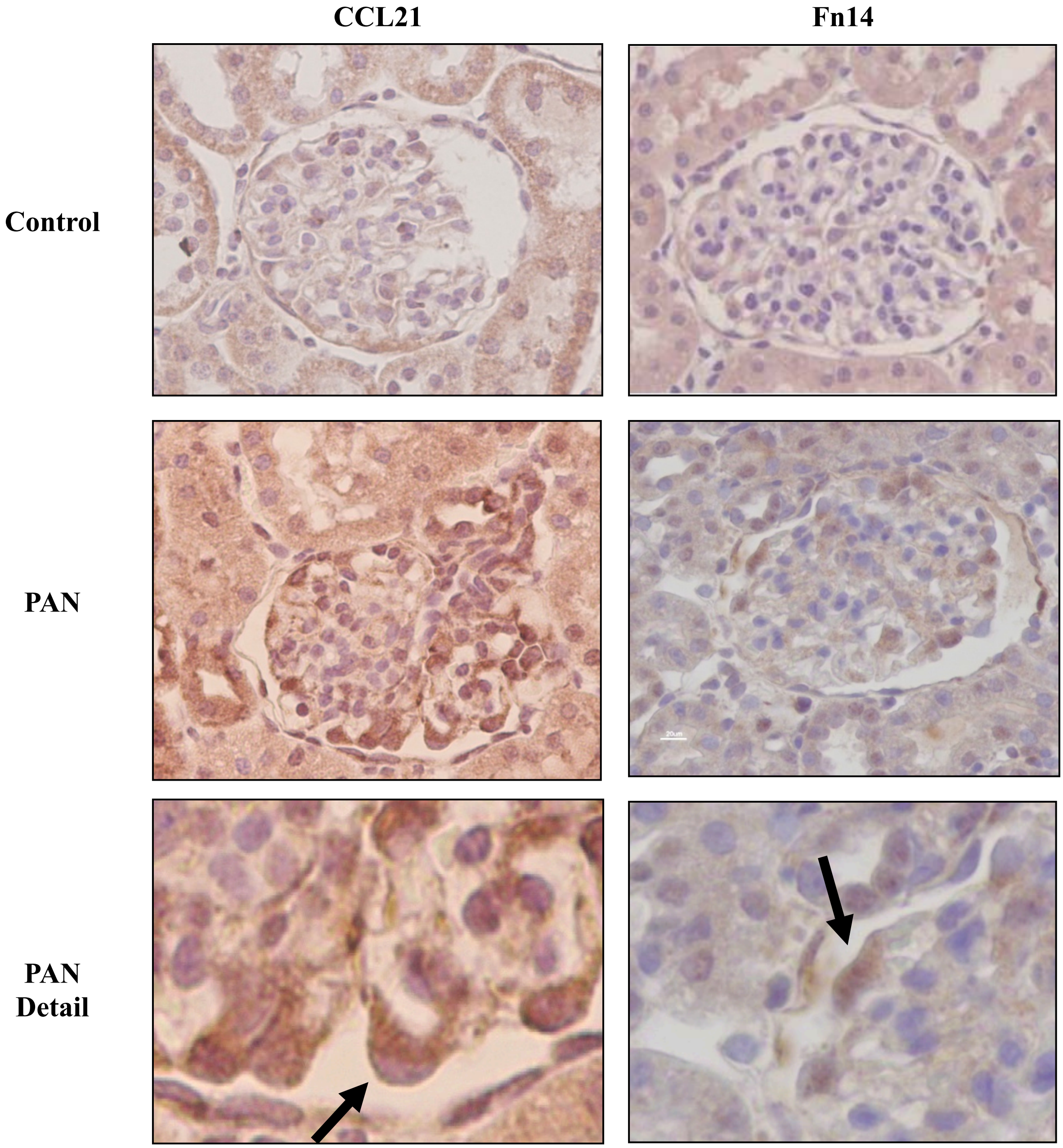

Supplementary figure 2. Fn14 and CCL21 upregulation in PAN nephrosis. Immunohistochemistry. Fn14 and CCL21 immunohistochemistry 10 days following PAN or vehicle injection. Note podocyte expression of Fn14 and CCL21 in PAN-treated rats (arrows). Original magnification x400. Detail x1000. Images representative of 6/7 animals per group.

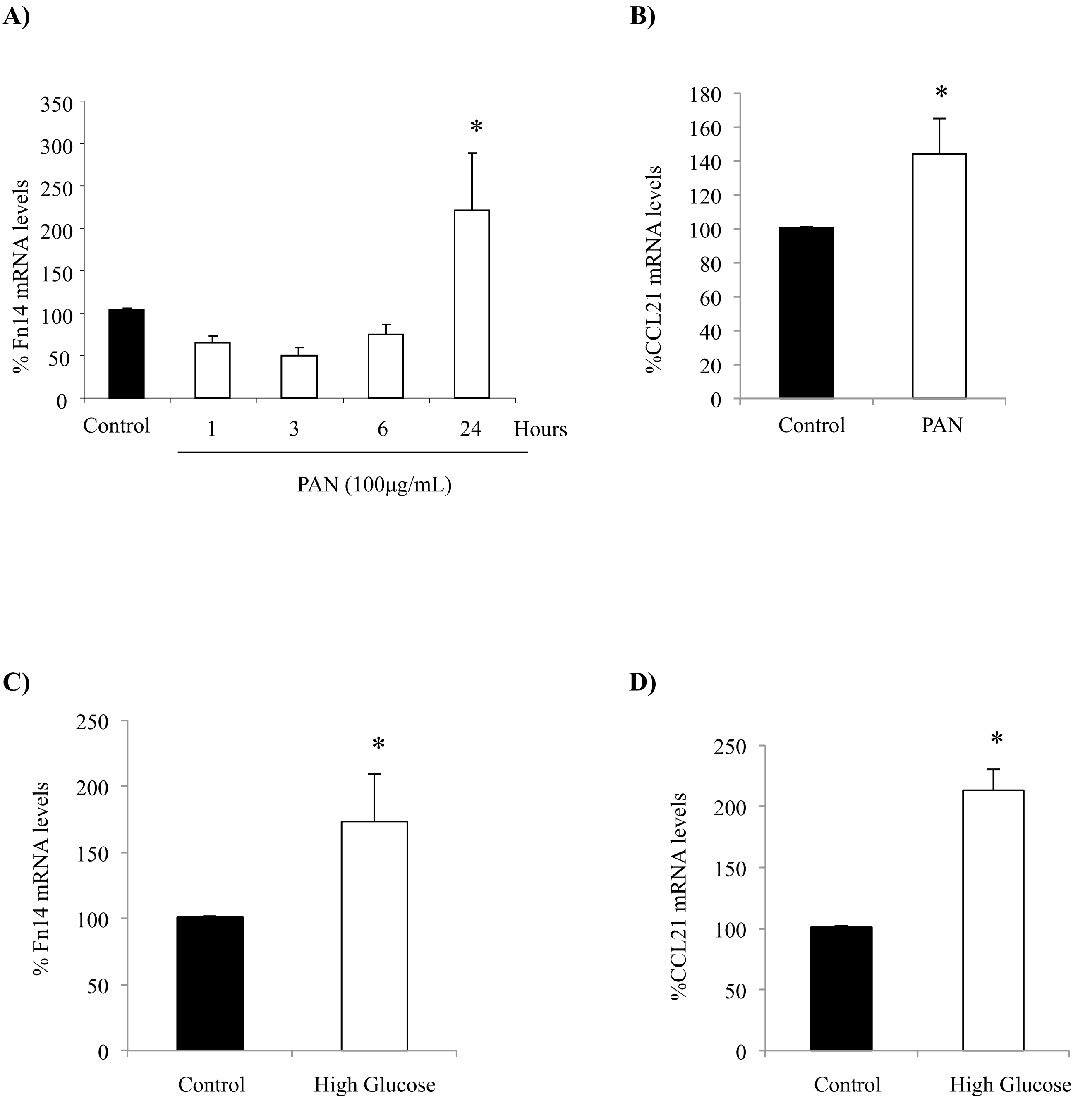

Supplementary figure 3. Impact of podocyte injury on Fn14 and CCL21 expression. Podocytes were cultured in presence of a cytotoxic concentration of PAN (100 µM) for up to 24 hours or high glucose for 24 hours. **A,C)** Fn14 mRNA and **B,D)** CCL21 mRNA. Real time RT-PCR. Mean±SD of 3 independent experiments. *p<0.01 vs control.
